# To Rich Christian Men

**Published:** 1854-03

**Authors:** 


### TO RICH CHRISTIAN MEN.

THE object of this article is to induce you to send five, ten, or a hundred dollars apiece, to enable us to furnish the JOURNAL to theological students and clergymen for one year; it will be economy to the Church of more than money can buy—the health and life of your ministers. The following are the considerations offered:—

As a very general rule when a man gets sick it is his own fault—the result of either ignorance or presumption.

No minister has a right to do a thing which has destroyed the health of others. This Journal will give facts, from time to time, which will show how health has been lost; and forewarned is to be forearmed.

A clergyman becomes more valuable to the Church every additional year of his life, from his increased experience, wisdom, personal forbearance and kindliness toward all his race, up to the full age of fourscore years and over. But scarcely a week passes without bringing to our knowledge the name of some eminent or useful preacher, who has given up his charge on account of ill health—some of them at the early age of thirty years.

It is no uncommon thing to hear of young men, " the hope of the Church," leaving the " seminary" on account of ill health.

Multitudes of instances have occurred where young men of unusual promise have entered the ministry, only to die at the threshold. All over the land are clergymen, who have gone down from their great work on account of ill health, and are making a painful and precarious living by keeping store, teaching school, working on a farm, or, worse still, existing on in heart-eating idleness, because they can find nothing they " can turn their hands to," while their children are growing up in neglect, and want, and pinching poverty. Who can measure the depth of that heart-gnawing which an educated and refined father must endure, who contemplates the sad sight from month to month and—hopelessly.

It possesses high advantages for ministers to get sick in the city—a substitute is secured, salary is continued, and a purse of a thousand or two is made up to defray a year's expense abroad; but such is not the happy lot of the country clergyman. His salary at most, when regularly, and promptly, and fully paid, is barely enough to meet the actual necessities of his family. When he becomes disabled, he has nothing laid up for a rainy day, and as he cannot perform his duties, his salary is as certainly stopped as the wages of a day-laborer on a ditch or a railroad; and then comes the want, the fearful struggle, and the terrible crushing-up of hope and ambition, literally terrible—how terrible none can so well know as the physician, to whom these tales are told so often by the strong man in tears. The Editor's office and correspondence can bear witness to many scenes like these, and he has been led thereby to attempt a preventive,—and that is, the publication of this Journal, which shall instruct young gentlemen at the "seminary" and clergymen "in the field" how such disasters are brought about, and knowing them rightly, that is practically, is to avoid them: thus—

I once knew a clergyman of some fifty-five years, of singularly retiring manners; he was a worker, and he had a great mind; he commanded the best pulpits in the State, and he had no superior there. On one occasion he preached with his usual earnestness in a warm room in cool weather, and perspired freely; it was in a country church, at night. After the services he went home with one of the members, and was soon shown to the "prophet's chamber;" he discovered the sheets were a little damp, that the room and bedding seemed to have been unused for some time; there was a rawness in the atmosphere of this apartment which was exceedingly unpleasant, but not wishing to give trouble, he retired; the perspiration was immediately checked, he became thoroughly chilled, and died the next day in unutterable agony. His last words were, "I can't bear this pain long." Can any clergyman read this, and place himself in damp sheets within an hour after preaching a sermon with energy enough to dampen his inner garments by the perspiration from his person? Such a man thus instructed would bear the curse of a suicide.

Another clergyman, second to none in this wide land, rises at four every morning the year round, and from the perfect darkness of six or eight hours' sleep exposes the eyes to the glare of artificial light on a white page, and perseveres in it, against the advice of his physician. The result is an impairment of the sight, a cessation from all ministerial labor, a useless journey abroad, the giving up of his church, with that abiding depression, that deep despondency which a good man feels, who is thus told before the heat and burden of the day is past, that his work is done. Some three years have thus passed away, and whether he will ever be restored, time only can tell. Has any sane man, after learning this fact, any right to suppose that his eyes are so much better than others' that they can endure such use with impunity? Not one man in a thousand can thus use them. Any man of common sense ought to have known, without the experiment, that such a course could not be otherwise than pernicious; but it is notorious that clergymen have not common sense. Somehow or other, they often become possessed with the idea that however any specified practice or thing may injure others, it would not injure them, and thus practically claim an exemption from the common laws of mortality. Is that right? What is a miracle? It is the suspension of a natural law for some special object. Has any creature a right to demand of his Maker the suspension of any of his laws, for his accommodation, that he may do silly things with impunity? I think not. This Journal, properly conducted, will be calculated to remedy these evils, and thus save millions of money to the Church; for when one clergyman leaves the ranks another must be educated, at an expense of at least three thousand dollars, to take his place, and when he does, he is nothing but a raw recruit, and alas! but too often, in consequence of inexperience, flounders, and falls into a lifelong inefficiency.

Money has been sent to me by practical Christian men, unsolicited, to pay for the Journal for their minister, and that circumstance has suggested this article, which is closed with the single statement, that twenty per cent. will be allowed on any money sent to me for the above purposes,—that is to say, for every hundred dollars, or other amount in proportion, one hundred and twenty Journals will be sent one year, to as many specified addresses; and if none are specified, it will be sent to clergymen in alphabetical order of the denomination of the donor.

Christian men, think of this. As soon as the publisher of the Journal is paid, the Editor will do more than any of you in this direction, he not being dependent on the Journal, by any means, for a living. It originated in a desire to preserve the health of theological students and clergymen, and was intended to be edited in the odds and ends of time which rising at five o'clock the year round would give from another calling; not indeed to read or write by candle light morning or evening, for this he has not done for twenty-four years, but to do other necessary things, which do not require any special strain on the eyes, such as attending lectures, sawing wood, mechanical contrivances, and above all romping and playing with the children, Nelly, and Molly, and little Bob, which, by the way, is the most delightfully glorious form of exercise imaginable, delightful to myself and them now, and mournfully pleasing to them, the reminiscence, in after years, when I have passed away.

Let me warn the reader here, in a separate paragraph, which will more than pay him for reading this article, never to take a step for the mere sake of exercise. To walk a mile to a post and then turn round and walk back again, must be the most tiresome of all tiring jobs. I speak from conjecture. I do not now recollect ever to have tried it. The reader might try it once, it may be a lesson, the memory of which would last through life, and be an inducement so to arrange things beforehand, that exercise might be connected with something agreeable and useful,—that is the kind which tells on the health, as no other can, to the hundredth part of the extent.

